# The importance of sex as a risk factor for hospital readmissions due to pulmonary diseases

**DOI:** 10.1186/s12889-019-8138-6

**Published:** 2020-01-14

**Authors:** Alessandra Buja, Anna De Polo, Elisa De Battisti, Milena Sperotto, Tatjana Baldovin, Silvia Cocchio, Patrizia Furlan, Mario Saia, Maria Luisa Scapellato, Guido Viel, Vincenzo Baldo, Chiara Bertoncello, Mark Ebell

**Affiliations:** 1 Department of Cardiological, Thoracic and Vascular Sciences, Hygiene and Public Health Unit, University of Padua, Via Loredan 18, 35128 Padova, Italy; 2 School of Specialization in Hygiene, Preventive Medicine and Public Health, University of Padua, Via Loredan 18, 35128 Padova, Italy; 3 ULSS 6 Euganea, Via Enrico degli Scrovegni 14, 35131 Padova, Veneto Region Italy; 4 Department of Cardiological, Thoracic and Vascular Sciences, Legal Medicine Unit, University of Padua, Via Loredan 18, 35128 Padova, Italy; 5 College of Public Health, University of Georgia, 125 Miller Hall, UGA Health Sciences, Athens, GA 30602 USA

**Keywords:** Pulmonary disease, Readmission risk, Sex differences, Elderly people

## Abstract

**Background:**

Pulmonary diseases are a common and costly cause of 30-day readmissions. Few studies have focused on the difference in risk for rehospitalization between men and women in older patients. In this study we analyzed the association between sex and the risk of readmission in a cohort of patients admitted to the hospital for chronic obstructive pulmonary disease (COPD) exacerbation and other major pulmonary diseases.

**Methods:**

This was a retrospective cohort study based on administrative data collected in the Veneto Region in 2016. We included 14,869 hospital admissions among residents aged ≥65 years for diagnosis related groups (DRGs) of the most common disorders of the respiratory system: bronchitis and asthma, pneumonia, pulmonary edema, respiratory failure, and COPD. Multilevel logistic regressions were performed to test the association between 30-day hospital readmission and sex, adjusting for confounding factors.

**Results:**

For bronchitis and asthma, male patients had significantly higher odds of 30-day readmission than female patients (adjusted odds ratio (aOR), 2.07; 95% confidence interval (CI), 1.11–3.87). The odds of readmission for men were also significantly higher for pneumonia (aOR, 1.40; 95% CI, 1.13–1.72), for pulmonary edema and respiratory failure (aOR, 1.28; 95% CI, 1.05–1.55), and for COPD (aOR, 1.34; 95% CI, 1.00–1.81).

**Conclusions:**

This study found that male sex is a major risk factors for readmission in patients aged more than 65 years with a primary pulmonary diagnosis. More studies are needed to understand the underlying determinants of this phenomena and to provide targets for future interventions.

## Background

Countries with developed healthcare systems are working to reduce unnecessary hospital readmissions to reach the triple aim of reducing costs, improving patient satisfaction, and improving health. Readmissions impact both health and satisfaction of patients, as multiple hospitalizations of subjects with chronic comorbidities are associated with emotional distress, loss of function, nosocomial infections, and increased mortality [1]. Moreover, hospital readmissions are also considered an index of low-quality care and in some health systems incur financial penalties for hospitals [2]. It is therefore important to analyze which factors lead to readmissions and to develop efficient strategies to reduce unnecessary readmissions, both at the hospital and health system levels.

Among the top 20 causes of readmission within 30 days are several respiratory diagnoses, including respiratory insufficiency and respiratory arrest (3rd place), COPD and bronchiectasis (9th place), and aspiration pneumonia (11th place) [3]. While several studies have analyzed the risk factors for rehospitalization in patients with pulmonary disease, few have focused on the difference in risk between men and women. In this study we analyzed data from a cohort of patients from the Veneto Region to determine the association between sex of the patient and readmission to the hospital. The ultimate goal is to use this information to help tailor a discharge program to prevent readmission and waste in resource use.

## Methods

In the Veneto Region, regional authorities coordinate and control local health units (LHU), each of which is a separate unit in the Italian National Health Service (NHS). The NHS plans and delivers health services, primary care, and hospital care to its local community, based on a regional health plan. This was a retrospective cohort study conducted in any facility operating under the NHS in the Veneto Region in 2016 (population 4,907,529).

In order to select the “Index Admissions” (IA), we included diagnosis-related groups (DRGs) of common respiratory diagnoses involving residents aged ≥65 years: bronchitis and asthma (DRG 96 and 97); pneumonia (DRG 89 or 90); pulmonary edema and respiratory failure (DRG 87); and COPD (DRG 88). We excluded patients meeting the following criteria: admission with International Classification of Diseases 9th revision clinical modification (ICD-9-CM) principal diagnostic codes cancer (140._-239._), admission with psychiatric DRG (425–433 and 523), admission with DRG of chemotherapy and radiotherapy (409, 410, 492), admissions for day hospital care or rehabilitation, and those concerning patients residing outside the region. We also excluded as IA whose discharge was due to voluntary discharge, transfer to another public or private care institution for acute cases, transfer to another ward of the same structure, or transfer to a rehabilitation institute. If the same patient was admitted to hospital for the same condition several times, all the admissions were considered as IA. “Hospital readmission” was defined as the same patient readmitted for the same disease within 30 days of the IA.

Multilevel logistic regression models were created with hospital readmissions within 30-days for each pulmonary condition above as the dependent variable (attributing a value of 1 for each indicator associated with a readmission, as defined earlier) and considering the sex as the independent variable. Potential confounders included at first admission level: age as a continuous variable, formal education (university, high school, middle school and no education/primary school), citizenship (Italian/Not Italian), length of stay as continuous variable, type of discharge (at home, at home with domiciliary care, residential care), and the Charlson Comorbidity Index (CCI) as a continuous variable; at second level the type of care institute (university, not university). The Charlson Comorbidity Index measures the comorbidities inside each hospital admission and is an extensively used comorbidity index with predictive validity for a range of outcomes, including readmission and death. The CCI comprises 19 medical conditions weighted 1–6 on the basis of their association with mortality.

Statistical analyses were performed using STATA software, version 12.1. All *p*-values reported are two-sided and results with *p*-values below 0.05 were considered statistically significant.

## Results

During the period considered, we identified 1140 admissions for bronchitis and asthma, 6258 admissions for pneumonia, 5260 admissions for pulmonary edema and respiratory failure and 2211 admissions for COPD, collected in the Veneto Region in 2016.

The sample’s characteristics are shown in Table 1. Table 2 shows the number of Index Admissions and the percentage of readmissions overall and by sex for selected respiratory conditions: the frequency of readmission was statistically higher for men in all the considered cases.

**Table 1:** Characteristics of adults aged 65 years and older years hospitalized for selected pulmonary diseases in 2016, Veneto region. (SD: standard deviation)

		Bronchitis and asthma (*N* = 1140)	Pneumonia (*N* = 6258)	Pulmonary edema and respiratory failure (*N* = 5260)	COPD (*N* = 2211)
Gender	Male	445	3220	2580	1152
	Female	695	3038	2680	1059
Age	65–84 yr	624	3407	3323	1378
	Over 84 yr	516	2851	1937	833
Citizenship	Italian	1124	6215	5191	2185
	Other	16	43	69	26
Formal education	University	17	98	74	33
	High school	48	319	258	112
	Middle school	194	1102	996	413
	Primary school or none	881	4739	3932	1653
Charlson Comorbidity Index	No comorbidities	645	2723	1255	1124
	At least one comorbidity	495	3535	4005	1087
Type of care institute	University hospital	92	753	632	257
	Other	1048	5505	4628	1954
Length of stay in the care institute	(mean ± SD)	8,49 ± 4,98	11,66 ± 7,13	12,55 ± 7,80	9,59 ± 5,80
Type of discharge	Home	1029	5642	4714	2036
	Home with domiciliary care	51	300	142	104
	Residential care	60	496	404	71

**Table 2:** Number of index admissions and % of readmissions overall and by sex for selected pulmonary diseases in adults age 65 years and older in 2016, Veneto Region

	Total		Male		Female	
	IA	% R^a^	IA	% R^a^	IA	% R^a^
Bronchitis and asthma	1140	4.1	445	5.6^†^	695	3.2
Pneumonia	6258	6.5	3220	7.3^†^	3038	5.7
Pulmonary edema and respiratory failure	5260	9.5	2580	10.4^†^	2680	8.6
COPD	2211	9.5	1152	10.8^†^	1059	8.1

^a^*R* readmissions (patients hospitalized again for the same disease within 30 of their IA)

^†^*p* < 0.05 (vs female)

Table 3 shows the results of the multilevel logistic regression analyses. For bronchitis and asthma, male patients showed twofold higher odds of being readmitted than female patients (aOR, 2.07; 95% CI, 1.11–3.87). Significant associations with sex also emerged for pneumonia (aOR, 1.40; 95% CI, 1.13–1.72), for pulmonary edema and respiratory failure (aOR, 1.28; 95% CI, 1.05–1.55) and for COPD (aOR, 1.34; 95% CI, 1.00–1.81).

**Table 3:** Gender as a risk factor of readmission for selected pulmonary diseases in adults aged 65 years and older, by multivariate logistic regression analysis

Disease	Odds ratio (95% confidence interval) Male vs Female
Bronchitis and asthma	2.07 (1.11–3.87)
Pneumonia	1.40 (1.13–1.72)
Pulmonary edema and respiratory failure	1.28 (1.05–1.55)
COPD	1.34 (1.00–1.81)

Adjusted for: age, formal education, citizenship, length of stay, Charlson Comorbidity Index, type of discharge, type of care institute

Results in bold are statistically significant (*p* < 0.05)

## Discussion

This population-based study found that male sex is a risk factor for readmission in patients aged more than 65 years admitted to hospital with the diagnosis of several common respiratory diseases: bronchitis and asthma, pneumonia, pulmonary edema and respiratory failure and COPD.

This sex gap has been reported previously in other studies conducted in various settings, where male sex is indicated as an independent risk factor for readmission for COPD [4, 5]. Dal Negro [6], analyzing Italian patients diagnosed with COPD, chronic bronchitis and emphysema, concludes that direct costs for management are higher in male than in female patients. Moreover, this study draws attention to the fact that the major part of direct costs for the management of this diseases is yielded by the inpatient hospitalization. As regards pneumonia, our findings are concordant with literature data, which shows that women have less risk to be readmitted for pneumonia than men [7].

This relevant phenomenon is difficult to explain clinically but could be due to management of disease after discharge. In fact, men report less help-seeking behavior, which may delay accessing care when it is needed [8]. Moreover, men use primary care health services less frequently than women, are less involved in preventive initiatives and are less health literate [9]. For instance, it has been seen that fewer men understand and attend their follow-up appointments after acute hospitalization compared with women [10].

Help-seeking behavior is a complex phenomenon in which gender plays a fundamental role.

Masculine attitudes, behavior and values in general could lead to men ignoring symptoms of ill-health and failing to seek help from the health services because they see it as a sign of weakness [11]. Risk-taking behaviors are also more strongly associated with male role models [12]: in many cultures, the use of tobacco (a major risk factor for several lung diseases and associated with their severity) is closely linked with the perception of being a “real man” [13]. Some biological factors may also influence the higher risk of rehospitalization for pneumonia in men. For instance, men have a weaker immune response and have also been shown to have more chronic mucus hypersecretion, which may worsen their prognosis and increase the likelihood of death [7]. The association of sex with post-hospitalization risk is complex, and likely to be influenced by multiple factors. However, whatever the causes of the phenomenon, it is important to think about how to prevent readmissions.

Indeed, it could be useful to talk about gender-based medicine and prevention strategies, in order to address the resources in the most efficient way to reduce readmission rate and, consequently, costs for the health system.

To achieve this goal, it is important to give male patients adequate access to providers and personnel of intermediate and long-term care plans [14].

In particular, currently, there are few studies that evaluate the effectiveness of interventions that promote the access of men to primary care. A recent review found that physical activity, education, peer support-based interventions improve quality of life in men with long-term conditions [15]. More studies are needed to understand what is successful in improving elderly men’s health and reducing the risk of readmission.

This study relied on routinely-collected administrative data, which unfortunately provides no information on the severity of a patient’s disease, which is a variable strongly associated with the probability of readmission. In this context, length of stay during the first hospitalization could serve as a vague “proxy” of this aspect, and it was used as possible confounder.

## Conclusions

This study found that male sex is a risk factors for readmission in patients aged more than 65 years with several pulmonary diseases.

More studies taking sex perspective into account are needed in order to provide targets for patient management interventions.

## Data Availability

All relevant data are within the paper. Requests for additional information should be addressed to the corresponding author and data may be provided on reasonable request.

## Abbreviations

aOR: adjusted odds ratio
CCI: Charlson Comorbidity Index
CI: Confidence interval
COPD: Chronic obstructive pulmonary disease
DRG: Diagnosis related group
IA: Index admission
ICD-9-CM: International Classification of Diseases 9th revision clinical modification
LHU: Local Health Unit
NHS: National Health Service
R: Readmissions
SD: Standard deviation
